# The Role of Low-Dose Quetiapine in the Treatment of Somatic Symptom Disorder: A Case Report

**DOI:** 10.7759/cureus.50116

**Published:** 2023-12-07

**Authors:** Shayranisse Pagan Cruz, Patricia M Guiribitey, Ginger Shupe

**Affiliations:** 1 Psychiatry, Larkin Community Hospital, Miami, USA

**Keywords:** cognitive behavioural therapy, selective serotonin reuptake inhibitor, second generation antipsychotics, serotonin norepinephrine reuptake inhibitor, somatic symptom disorder

## Abstract

Somatic symptom disorder (SSD) involves physical symptoms like palpitations, pain, weakness, dizziness, and pseudo-neurological symptoms. These symptoms are accompanied by excessive thoughts, emotions, and behaviors related to the symptom, causing significant distress and impairment lasting at least six months. They may not be explained by any underlying medical conditions. SSD can be resistant to standard treatment modalities like Cognitive Behavioral Therapy (CBT), Selective Serotonin Reuptake Inhibitors (SSRIs), and Serotonin and Norepinephrine Reuptake Inhibitors (SNRIs). Antipsychotics, in particular second generations, have also been used to treat SSD but not as frequently. This case report shows the improvement in symptomatology of SSD after low-dose quetiapine was used for its management. This case is a 41-year-old Hispanic male with a diagnosis of SSD who presented to the outpatient clinic for severe somatic symptoms. The use of low-dose second-generation antipsychotic (SGA), in particular quetiapine, to successfully improve symptoms and patient functionality after just four weeks on quetiapine as the patient and wife both reported significant normalization of intrusive thoughts and health-related behaviors. To the best of our knowledge, this is the first case demonstrating symptom improvement in SSD following the addition of low-dose quetiapine to SNRI treatment.

## Introduction

Somatic Symptom Disorder (SSD) is identified when an individual displays a significant preoccupation with physical symptoms, such as pain, weakness, or difficulty breathing, resulting in substantial distress and functional impairment lasting at least six months [1]. The person exhibits excessive thoughts, emotions, and behaviors related to these physical symptoms. The presence or absence of a medically diagnosed condition is not the sole determining factor for the diagnosis. The key focus lies in the degree to which the thoughts, emotions, and behaviors associated with the illness are excessive or disproportionate [1]. It is crucial to note that the patient genuinely believes they are unwell and is not feigning the illness [1].

While the etiology of SSD is unclear, studies have linked SSD to some risk factors such as childhood neglect, sexual abuse, chaotic lifestyle, and history of alcohol and substance abuse. Moreover, severe somatization has been linked to personality disorders, specifically avoidant, paranoid, and obsessive-compulsive personality disorders [2]. Psychosocial stressors, such as unemployment and impaired occupational functioning, have also been implicated. The underlying mechanisms of SSD are not yet fully understood. However, it is hypothesized that autonomic arousal resulting from endogenous noradrenergic compounds may contribute to symptoms such as tachycardia, gastric hypermotility, heightened arousal, muscle tension, and pain associated with increased muscular activity in individuals with SSD [2]. Additionally, there may be a genetic component involved. A study examining monozygotic and dizygotic twins revealed that genetic factors accounted for 7% to 21% of somatic symptoms, while environmental factors accounted for the remaining portion [2]. Another study identified various single nucleotide polymorphisms associated with somatic symptoms [2]. SSD is believed to affect approximately 5% to 7% of the overall population, with a higher occurrence among females (with a female-to-male ratio of 10:1) [3]. It can manifest during childhood, adolescence, or adulthood [3]. The prevalence increases to approximately 17% of the primary care patient population [3]. The prevalence is likely higher in certain patient populations with functional disorders, such as fibromyalgia, irritable bowel syndrome, and chronic fatigue syndrome [3]. It is rare to encounter patients with SSD.

The gold standard for the management of SSD is psychotherapy, with Cognitive Behavioral Therapy (CBT) showing significant improvement in patient functionality. However, somatic symptom disorder can be refractory to even CBT and pharmacological treatment with Selective Serotonin Reuptake Inhibitors (SSRI) and Serotonin and Norepinephrine Reuptake Inhibitors (SNRI) [3]. Antipsychotics, in particular second generations, have been gaining favoritism lately as they are being used as adjuncts to SSRI and SNRI to manage SSD [2]. This case is a 41-year-old Hispanic male who presented to the outpatient clinic for severe somatic symptom disorder. We discussed the use of a low dose of quetiapine, a Second-Generation Antipsychotic (SGA), to successfully improve SSD symptoms. Quetiapine primarily exerts its effects by blocking dopamine D1, D2, histamine H1, alpha-1, and alpha-2 adrenergic, as well as serotonin types 1 and 2 (5-hydroxytryptamine (5HT)1A and 5HT2) receptors. Quetiapine is FDA-approved for the management of schizophrenia, bipolar I mania and depressive episodes, bipolar maintenance, and as an adjunct to antidepressants for Major Depressive Disorder (MDD) [4]. However, it is not FDA-approved for monotherapy or as an adjunct in SSD. A study conducted in China explored the off-label use of antipsychotic medications in psychiatric patients, excluding those with schizophrenia spectrum disorder or bipolar disorder [3]. The findings revealed that antipsychotics were utilized across a broad range of psychiatric conditions, with the highest usage observed in patients with dissociative and/or conversion disorders, as well as other mental disorders, including somatoform disorders, major depressive disorder, anxiety disorder, and insomnia [5]. The most frequently employed antipsychotics were olanzapine (29.1%), quetiapine (20.3%), and risperidone (6.8%) [3]. Augmentation with atypical antipsychotics such as paliperidone and aripiprazole has shown promising results, particularly in cases of treatment-resistant SSD [3].

A literature search was conducted for our case report on PubMed, EMBASE, and COCHRANE from January 1st, 2013, to May 2023 using the keywords "somatic symptom disorder", "second generation antipsychotics", "atypical antipsychotics", "selective serotonin reuptake inhibitors", "serotonin-norepinephrine reuptake inhibitors", "primary care physician" and "cognitive behavioral therapy." Publications found through this indexed search were reviewed and manually screened to identify relevant studies.

## Case presentation

The patient presented in our clinic was a 41-year-old male who exhibited severe somatic symptoms accompanied by high distractibility to his body surfaces and feelings of restlessness. The patient also exhibited current severe depressive symptoms, anxiety, and insomnia. He has no past medical history and has a prior psychiatric diagnosis of major depressive disorder. Our patient was born and raised by his parents and comes from a Hispanic family. He has been married for over a decade and has three children, with the two youngest being diagnosed with Autism Spectrum Disorder (ASD). The patient did not have a history of trauma or history of substance use disorder. He completed a Bachelor's Degree and was a small business owner, which he has successfully run until now. The patient had his first depressive episode 20 years ago when he received the diagnosis of MDD and was started on pharmacological treatment with paxil, which was continued for one and a half years until remission was achieved. At that time, he didn’t experience associated anxiety and/or somatic symptoms. The patient also did not experience psychotic symptoms in the last depressive episode and has never required psychiatric hospitalization. The recurrence of depressive symptoms, insomnia, and anxiety began eight months prior to his first encounter, which he attributed mainly to his second child's ASD diagnosis.

Due to a significant functionality decline, his wife brought him to the clinic. The severity of the somatic symptoms was significant for lumbar back pain, which he perceived as renal failure. The patient also had perseveration with a significant focus on gastrointestinal symptoms, such as constipation, indigestion, and abdominal bloating, with circular thoughts related to a false belief of unemptied bowels, even though his wife showed him evidence of his healthy stool pattern. He engaged in long-lasting rituals of misuse of laxatives, multiple visits to the bathroom, and a significant decrease in oral intake, which led to a 30-pound weight loss over four months. Another somatic preoccupation included a false belief of an unemptied bladder, which made the patient convinced of having a diagnosis of Neurogenic Bladder. In the months before the patient's first visit to the clinic, he visited the emergency department multiple times with subsequent referrals to specialists, including Internal Medicine, Neurologist, Urologist, Gastroenterologist, and Nephrologist. All investigations and examinations, including but not limited to Brain and Spine Magnetic Resonance Imaging, Electromyography, Abdomino Pelvic Computed Tomography with and without contrast, Electrocardiogram, and laboratory workup (thyroid, renal, and liver function), all came back with unremarkable findings. Despite reassurance from specialists, the patient continued with constant anxiety about his symptoms for eight months, leading to further unnecessary exams and follow-ups. The severity and impact of physical symptoms have caused functional impairment affecting his daily living, including poor performance at his job and with his family. 

Three months before his current outpatient psychiatric clinic, he was evaluated by his primary care physician (PCP). Initially, the patient’s complaint was insomnia, for which he was treated with 50 mg of trazodone; however, it was ineffective. The patient proceeded to complain of worsening insomnia, somatic symptoms, and anxiety. His PCP then started him on 25 mg of sertraline and 0.5 mg of Xanax® as needed daily but the patient reported worsening somatic symptoms and self-discontinued it four days before his presentation to our clinic. The patient had been receiving CBT weekly for the past two months without significant improvement. To this point, the severity of somatic symptoms increased abruptly. Upon the first approach, the patient was internally preoccupied and requested an immediate transfer to the Emergency Room on multiple occasions throughout the evaluation. He admitted the severity of the symptoms and functional impairment has led to suicidal ideations with a plan of jumping from a building but denied current intent. He was reluctant to restart sertraline or any other SSRI due to gastrointestinal side effects, which exacerbated his somatization. It was decided to start the patient on an SNRI (venlafaxine XR 75 mg orally daily) along with a low dose of an SGA (quetiapine 50 mg orally at bedtime). The patient was re-evaluated one month later and reported total adherence to treatment. Upon reevaluation, the patient showed overall significant improvement in somatic symptoms along with decreased anxiety and improved mood. He still experienced some somatic symptoms but with less severity, such as low back pain. He only visited the emergency department once monthly, whereas before, it had been daily, reflecting a better insight into his diagnosis. The patient reported concerns about bowel movements, which were reduced from six hours to approximately one hour, with associated improvement in abdominal bloating, constipation, and indigestion, leading to a secondary nine-pound weight gain in one month. The patient also reported significant improvement in mood, sleep, energy levels, and motivation to complete daily tasks and denied anhedonia, hopelessness, and suicidal ideations. During subsequent monthly follow-ups, the patient only reported “blurry vision” and no longer reported back pain, paresthesias, dizziness, or constipation. He hasn’t been observed restless since his first evaluation and has significantly recovered his functionality by resuming work and being able to help his wife with the care of their children and house chores.

## Discussion

It has been well established that SSD can be detrimental to a patient's well-being and causes a significant negative impact on functionality. Extensive data has demonstrated that in routine clinical settings, the prevalence of somatic symptoms and disorders is progressively escalating, contributing to significant disability and distress among individuals [6]. Patients suffering from SSD usually seek psychiatric care only after having undergone many different ineffective medical assessments and diagnostic examinations. Individuals with somatic symptom disorder (SSD) typically seek psychiatric care after undergoing many different unsuccessful medical assessments and diagnostic examinations. SSD treatment represents a difficult challenge to psychiatrists due to the delay in patients receiving proper evaluation and management. This case study demonstrates the efficacy of a low dose of quetiapine as an adjunct to SNRI in the treatment of severe SSD by improving preoccupation associated with somatic symptoms, along with full recovery of the patient's functionality in different domains of his life. Additionally, we proposed the consideration of a low dose of quetiapine to treat SSD instead of benzodiazepines as an adjunct to antidepressants.

Benzodiazepines are frequently prescribed for anxiety disorders and are widely used. While clinical practice guidelines recommend their short-term adjunctive use, previous research has highlighted concerns regarding the long-term usage patterns of benzodiazepines [7]. This prolonged use poses problems due to side effects, potential dependence, and abuse risks. Some potential side effects associated with their extended use include sedation, cognitive impairment, and impaired psychomotor performance [6]. For instance, a Cochrane review that examined nine randomized controlled trials involving 679 subjects (n = 697) demonstrated that the combination of antidepressants and benzodiazepines was more effective than antidepressant monotherapy in terms of efficacy outcomes after one and four weeks of treatment [8]. However, this superiority was not observed for treatment durations of six weeks or longer [8].

As described above in the case presentation, the patient had severe somatic symptoms with excessive worries and extreme impairment in functionality. This was successfully treated by adding a low dose of quetiapine as an adjunct to an SNRI. Considering the challenge that represented starting any antidepressants in the treatment of SSD due to exacerbations in anxiety and somatic symptoms, a low dose of quetiapine and other SGA may improve adherence and effectiveness of treatment [3]. By the end of the four-week treatment involving a combination of quetiapine and venlafaxine, positive outcomes were observed in terms of decreasing depressive and anxiety symptoms, as well as normalizing disrupted thought contents and related behaviors. Second-generation antipsychotics (SGAs), specifically quetiapine, olanzapine, and risperidone, have shown potential to improve symptoms for patients who have an inadequate response to antidepressant monotherapy. However, it is recommended to use these medications in the short term due to the potential long-term side effects associated with their specific pharmacological profiles and interactions, including symptoms of tardive dyskinesia and metabolic issues such as hyperglycemia, weight gain, and hyperlipidemia.

Increasing evidence suggests that abnormal serotoninergic and noradrenergic neurotransmission play a significant role in somatic symptoms [9]. The physiological changes associated with impaired serotonin (5-HT) and norepinephrine (NE) signaling can lead to disruptions in signal transduction, decreased release of 5-HT or NE from presynaptic neuron terminals, alterations in receptor function and/or quantity, and modifications in intracellular signaling mechanism [9]. The concept of somatosensory amplification is believed to be highly significant in understanding the underlying mechanisms of somatization [9]. Research suggests that abnormal interactions within neural circuits involving large-scale systems responsible for visceral-somatic perception, emotional processing/awareness, and cognitive control play pivotal roles in the neurobiology of somatosensory amplification [10]. Key brain regions associated with somatosensory amplification include the anterior cingulate cortex, insula, amygdala, hippocampal formation, striatum, and several other regions [10].

Regarding the factors and neurobiological mechanisms underlying the effectiveness of low-dose quetiapine, its pharmacological profile appears to be a contributing factor. Quetiapine exhibits affinity towards multiple receptors, including D1, D2, histamine H1, alpha-1, and alpha-2 adrenergic, as well as serotonin types 1 and 2 (5HT1A and 5HT2), which is not found in first-generation antipsychotics [4]. Notably, it has a high 5HT2A/D2 receptor affinity ratio, indicating a stronger binding to 5-HT2A receptors compared to D2 receptors. Additionally, its partial agonism at 5HT1A receptors and antagonism at Alpha1 and H1 receptors contribute to increased sedative effects [4]. These pharmacological properties may explain the observed benefits of quetiapine in effectively alleviating severe somatic symptom disorder (SSD) in the patient. Notably, at sedative-hypnotic doses (e.g., 50 mg/day), the primary pharmacological property of quetiapine is its antagonism of histamine 1 receptors [4]. More research is needed to determine whether a low dose of quetiapine and its receptor affinity is useful for SSD treatment.

## Conclusions

Abnormal serotoninergic and noradrenergic neurotransmission are implicated in somatic symptoms, causing disruptions in signal transduction and receptor function. Somatosensory amplification involves neural circuit abnormalities in perception, emotion, and cognition. Quetiapine's multi-receptor affinity, including its high 5HT2A/D2 ratio, 5HT1A partial agonism, and Alpha1/H1 antagonism, may explain its efficacy in severe somatic symptom disorder. Utilizing low-dose quetiapine off-label could be a viable strategy in managing severe SSD, although further research is required to validate low-dose quetiapine's effectiveness in SSD treatment. Additionally, there is a need for specialized training and reliable diagnostic tools to enhance the early identification of SSDs.
